# An integrative analysis of DNA methylation and RNA-Seq data for human heart, kidney and liver

**DOI:** 10.1186/1752-0509-5-S3-S4

**Published:** 2011-12-23

**Authors:** Linglin Xie, Brent Weichel, Joyce Ellen Ohm, Ke Zhang

**Affiliations:** 1 Department of Biochemistry and Molecular Biology, University of North Dakota School of Medicine, Grand Forks, ND 58201, USA; 2 Bioinformatics Core, University of North Dakota School of Medicine, Grand Forks, ND 58201, USA; 3 Department of Pathology, University of North Dakota School of Medicine, Grand Forks, ND 58201, USA

## Abstract

**Background:**

Many groups, including our own, have proposed the use of DNA methylation profiles as biomarkers for various disease states. While much research has been done identifying DNA methylation signatures in cancer vs. normal etc., we still lack sufficient knowledge of the role that differential methylation plays during normal cellular differentiation and tissue specification. We also need thorough, genome level studies to determine the meaning of methylation of individual CpG dinucleotides in terms of gene expression.

**Results:**

In this study, we have used (insert statistical method here) to compile unique DNA methylation signatures from normal human heart, lung, and kidney using the Illumina Infinium 27 K methylation arraysand compared those to gene expression by RNA sequencing. We have identified unique signatures of global DNA methylation for human heart, kidney and liver, and showed that DNA methylation data can be used to correctly classify various tissues. It indicates that DNA methylation reflects tissue specificity and may play an important role in tissue differentiation. The integrative analysis of methylation and RNA-Seq data showed that gene methylation and its transcriptional levels were comprehensively correlated. The location of methylation markers in terms of distance to transcription start site and CpG island showed no effects on the regulation of gene expression by DNA methylation in normal tissues.

**Conclusions:**

This study showed that an integrative analysis of methylation array and RNA-Seq data can be utilized to discover the global regulation of gene expression by DNA methylation and suggests that DNA methylation plays an important role in normal tissue differentiation via modulation of gene expression.

## Background

With the exception of the transient wave of global demethylation that occurs during embryonic development, DNA methylation is considered, relative to covalent histone modifications, to be a more permanent, heritable mark. DNA methylation of cytosine at position C5 in CpG dinucleotides is reported to influence specific gene expression, especially to suppress gene expression [1]. CpG dinucleotides are largely depleted from the genome except for in regions referred to as CpG islands, found in the proximal promoter regions of many genes [2,3]. Most of these CpG clusters are unmethylated during normal cell development, except for tissue-specific differentially methylated genes [3], imprinted genes [4] and some X-chromosome inactivation related genes [5-7]. Modern genetic studies have established that DNA methylation is required not only for embryonic development [8], but also plays a critical role in diseased status such as cancer [9]. As the field of epigenomics expands to study multiple diverse normal and pathological processes, it becomes increasingly important to understand the role that normal global genome-wide DNA methylation patterns play in influencing global gene expression.

A differentiated cell in an organism is thought to contain essentially the same DNA as its ancestors, but it differs in the quality and quantity of gene expressions, and therefore functions differently. DNA-binding transcription factors are crucial determinants of gene expression, and many groups have shown that that epigenetic mechanisms such as nucleosome positioning and covalent chromatin modifications and are involved in regulating transcription factor accessibility and gene expression during cellular differentiation [10].

Understanding of DNA methylation in regulating normal tissue-specific genome function is still limited, although the causal relationship between DNA methylation and gene regulation has been well studied [10,11]. More than 150 tissue-specific differentially methylated regions (TDMs) have been identified via a Restriction landmark genomic scanning (RLGS) assay and expression of genes associated with these regions have been shown to be correlated with DNA methylation [12,13]. Other studies have linked DNA methylation to tissue-specific gene expression by studying the promoter regions of a small number of imprinted genes and those involved in maintenance of pluripotency [13-15]. Both sexes use genomic imprinting to control the expression of approximately 100 imprinted genes and allowing monoallelic expression from either the maternal or paternal allele, and most imprinted genes regulate placental and fetal growth [16,17]. However, studies on small subsets of tissue specific genes in mice could not prove an association between DNA methylation and gene expression [18,19]. The inconsistent result may result from a limited number of genes being studied and differences in the CpG island content of their proximal promoter regions.

The advent of global DNA methylation arrays and next-generation RNA sequencing transcriptome studies have made it possible to explore the global relationship between gene methylation and expression during cell development and tissue differentiation. Researchers using a comparative genomic approach to study the DNA methylation patterns among species have successfully identified a role of DNA methylation in the evolution of gene regulation across the tissues and species [20]. The fast development of next generation sequencing technology provides a comprehensive and reliable approach, RNA-Seq, to fast quantify the global transcription levels of a tissue [21]. RNA-Seq allows researchers to count the amount of mRNA being sequenced, and consequently, it more accurately measures the transcriptional levels than the fluorescent intensity-based methods, such as mRNA microarray and qPCR. In addition, RNA-Seq provides single nucleotide resolution for mRNA, therefore, it can differentiate gene isoforms (transcripts), whereas microarray is often not able to identify isoforms because it uses probes to detect mRNA levels. Methylation array and RNA-Seq techniques are powerful tools to study global methylation variation and transcription changes, but no joint analysis with these two type data have been reported yet. In this study, we use an integrative analysis of both data sets across three different normal human tissues with the intention of shedding light on understanding the underlying mechanism of DNA methylation mediating normal tissue differentiation.

## Results

The DNA promoter methylation data for human heart, kidney and liver tissue samples was obtained from the NCBI GEO database with the accession number GSE26033 http://www.ncbi.nlm.nih.gov/projects/geo/query/acc.cgi?acc=GSE26033. This data was generated by Pai et al. who studied the methylation patterns between human and chimpanzee tissues [20]. Six individual samples were collected for each tissue type. The methylation profiles were generated by Illumina HumanMethylation27 DNA Analysis BeadChip that contains 27,578 CpG loci located in either CpG islands or non-CpG islands of promoter regions.

The DNA methylation profiles of human heart, kidney, and liver were integrated with RNA-Seq data for analysis. The RNA-Seq data were obtained from Human BodyMap 2.0 project by Illumina, Inc. Human BodyMap 2.0 project conducted deep sequencing for total RNA of 16 individual human tissues with one sample for each tissue type. The RNA-Seq data was available in NCBI GEO database with accession number GSE30611 http://www.ncbi.nlm.nih.gov/projects/geo/query/acc.cgi?acc=GSE30611. In this study, we combined RNA-Seq data for heart, kidney and liver tissues with DNA methylation data.

### Data quantification and quality

**Figure 1:**
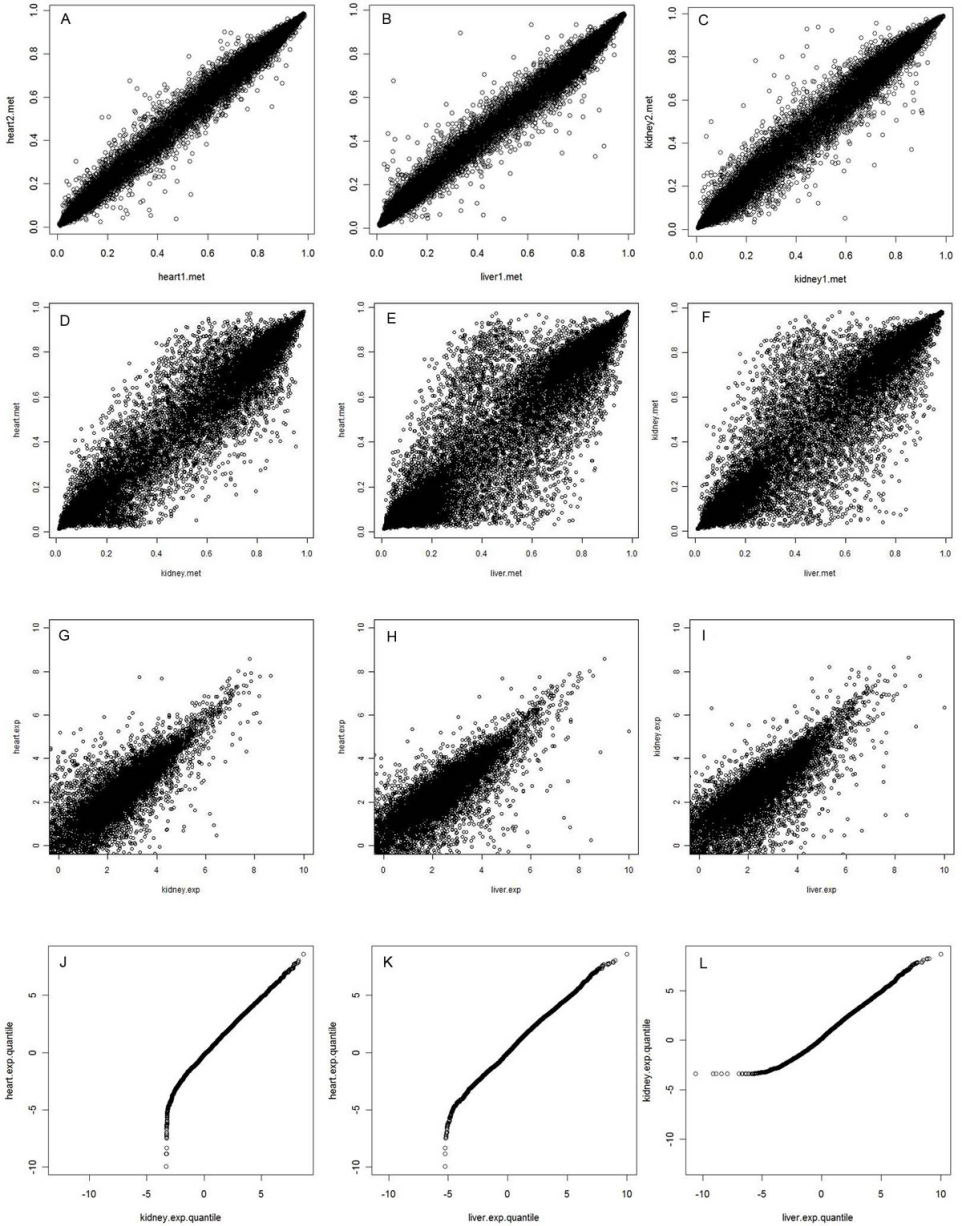
**Data quality and variation**. A-C. Plots of DNA methylation levels between a random pair of samples from the same tissues. D-F. Plots of average DNA methylation levels between the three tissues, heart, kidney and liver. G-I. Plots of RNA-Seq expression levels between the three tissues. J-L. QQ plots of RNA-Seq expression levels between the three tissues.

The variability in gene methylation level has various sources, including tissue differentiation, individual sample variation, and technical variance. For the same tissue, genes are expected to carry similar epigenetic modification to control tissue differentiation, thus the variability between sample replicates is expected to be marginal comparing with the variability between different tissues. To evaluate the quality of the DNA methylation data, we plotted the methylation levels between each pair of samples from the same tissue. All plots consistently showed low variability. Figure 1A-C show 3 plots for random pair of samples from heart, kidney and liver. The amount of genes that have a standard deviation less than 0.1 were 98.3%, 91.1%, and 97.6% for heart, kidney and liver, respectively. When we plot the average methylation level for each CpG marker between tissues, the variation was substantial larger (Figure 1D-F). It is clear that points in 1E and 1F are more widespread than that in 1D, implying difference between heart and kidney is smaller than that between heart and liver or between kidney and liver. Heart and kidney both develop from mesoderm and liver develops from endoderm, therefore, not only do epigenetic changes correctly differentiate between differentiated cell types, but they also reveal the distinct embryonic origins of tissues.

The RNA-Seq data from Illumina BodyMap 2.0 project was generated by Illumina HiSeq 2000 with 50 bp paired end sequencing. For each sample, ~160 million reads were generated. We used Tophat [22] to map short reads onto the reference sequence of human genome 19 (GRCh37/hg19). About 55% paired reads were aligned with the reference genome. Software Cufflinks [23] was then used to quantify the expression levels of transcripts. We counted from 45,000 to 48,000 transcripts from 3 tissues and compared the transcriptional levels between each pair of tissues. Figure 1G-I plot the pairwise expression levels between two tissues. The transcriptional levels are represented at logarithm scale for FPKM values calculated from Cufflinks. At log scale, the expression levels are aligned well between tissues with the majority of plots located in the lower level. To further explore the expression patterns, we drew quantile-quantile plots to compare the expression distributions between tissues (Figure 1J-L). All data distributions are aligned well in the high expression levels. However, at the lower end, there are dramatic differences between tissues. Heart has a number of genes with significantly lower expression than both kidney and liver, and liver has more low-expression genes than kidney.

### DNA methylation signature identifies tissue specificity

Figure 1D-E indicate variation in DNA methylation between tissues. Nonetheless, it is not clear whether the tissue-specific signatures can be identified from methylation data such that tissues can be classified based on DNA methylation pattern. We investigated sample classification for DNA methylation data with three different methods.

**Figure 2:**
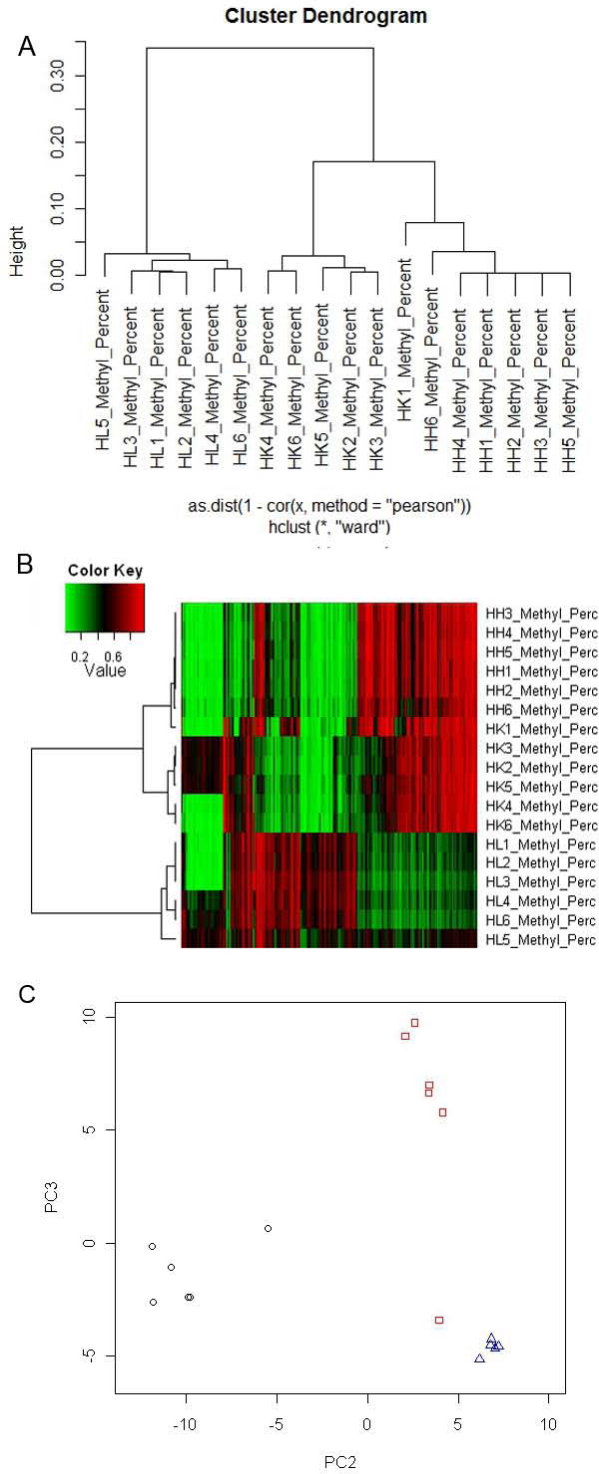
**Sample clustering based on DNA methylation data**. A. A dendrogram of hierarchical clustering of 18 tissue samples using all 27,578 DNA methylation markers. B. A heatmap of DNA methylation data with 488 CpG markers that have the highest standard deviation across all samples (sd > 0.2). Each row is a sample, and each column is a marker. A dendrogram by hierarchical clustering is given by hierarchical clustering. C. Methylation samples are plotted on the second and third principal components. The black circle points are liver samples, the red squares are kidney, and the green triangles are heart.

First, we performed hierarchical clustering using all 27,578 CpG markers. We tested different dissimilarity measures, including Euclidean distance, Pearson's dissimilarity and Spearman's dissimilarity. Pearson's and Spearman's dissimilarity are both based on data correlation, thus, they have similar performance that is better than Euclidean distance. For Illumina HumanMethylation27 data, most of genes have two or more CpG markers and the markers from the same genes should be correlated. Thus, correlation based dissimilarity method is more appropriate for this analysis. Ward linkage method was used in hierarchical clustering. The resulting dendrogram was shown in Figure 2A. The 18 methylation samples were clustered into 3 major groups, each representing a human tissue. Again, in terms of DNA methylation, heart and kidney are closer to each other than they are from liver. There was only one misplacement in this clustering, in that one kidney sample was clustered into heart group though it also has short distance to other kidney samples. Overall, tissue samples were successfully clustered based on their DNA methylation patterns.

Secondly, we looked for approaches to improve the hierarchical clustering result. The misplacement occurred in hierarchical clustering is likely due to the large number of markers. The markers with low variation should not contribute to tissue classification, but their variances may present as a noise to affect clustering. Therefore, we intended to filter out the markers with low variance prior to clustering. We selected only the CpG markers that have a standard deviation greater than 0.2 and we obtained 488 markers. A heatmap is shown in Figure 2B. Hierarchical clustering correctly classified all 18 samples with their tissue labels. In Figure 2B, green is for low methylation levels and red is for high levels. Most markers showed distinct methylation levels among the three tissues, therefore, they can be used as DNA signatures to represent different tissue. Note that the methylation levels of the first 69 markers in the heatmap are not aligned with tissue labels. We found that 68 markers are located in X chromosome, and they are methylation markers for sex imprinted genes. Their high levels (red) indicate female, and low levels (green) indicate male.

Thirdly, to further investigate methylation clustering, we used an alternative method, principal components analysis (PCA). One of the advantages of PCA is that it disassociates the correlation between markers when methylation data are transformed into principal components. We plotted the 18 samples in the first three principal components, and the samples are grouped into their tissue labels with the second and third principal components (Figure 2C). The circle points in black color are for liver samples, the red squares are kidney and the green triangles are heart. All points are grouped well except one kidney sample that is close to heart samples as in Figure 1A. However, from the dimension of principal component 2, this kidney sample is still in the range of other kidney samples rather than heart samples.

### Significantly expressed genes are affected by DNA methylation

**Figure 3:**
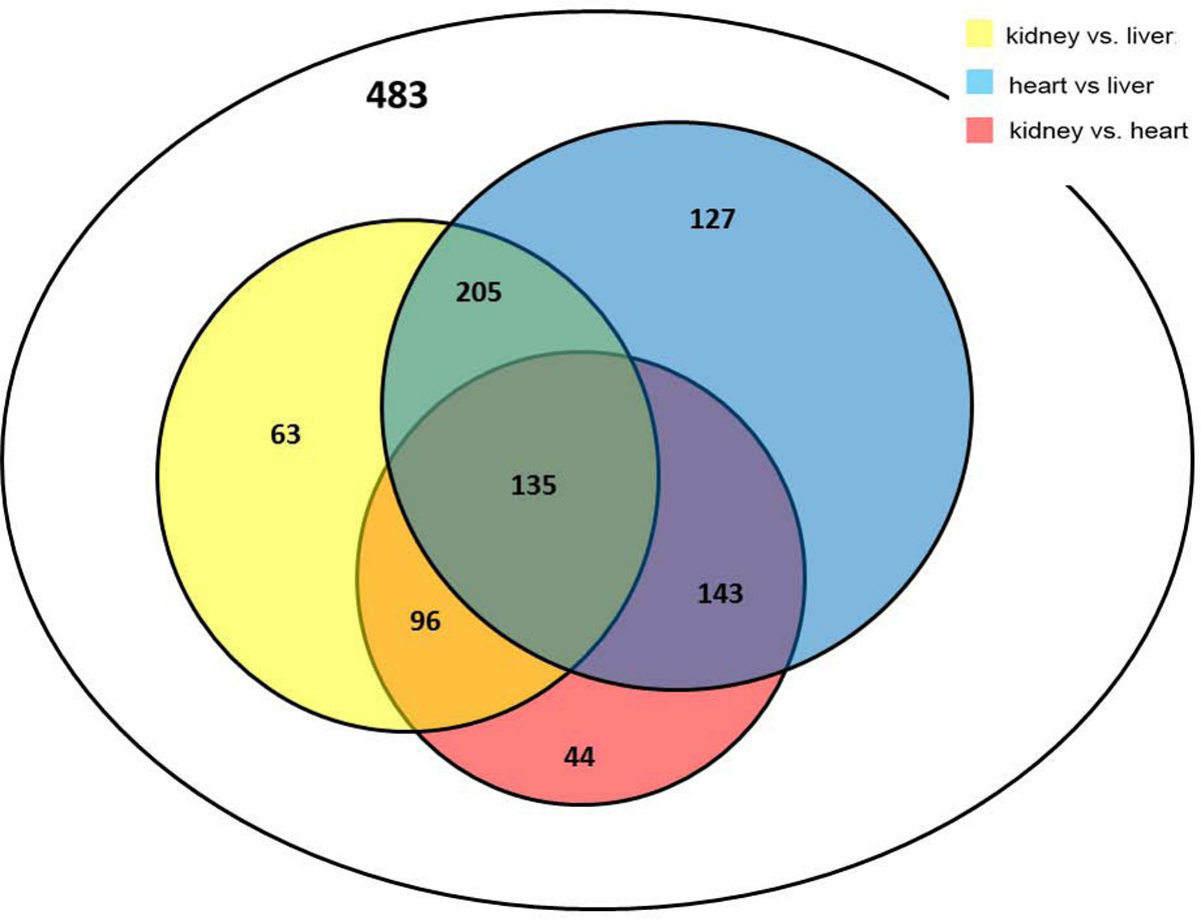
**A Venn diagram of differentially expressed genes**. Totally 1296 genes were differentially expressed between each pair of the three tissues. The numbers of genes that have significant methylation difference between tissues are given in this Venn diagram.

Next, we wanted to determine to what degree the gene expression differences among tissues are affected by epigenetic changes. We used cuffdiff to find expression variation (in FPKM) between tissues and then associated the selected genes with their methylation levels in HumanMethylation27 array. In total, we selected 1296 genes that were differentially expressed between any pair of the three tissues and that exist in HumanMethylatin27 array. We further looked at whether these 1296 genes showed DNA methylation variation between tissues as well. Unpaired t test was used to detect the methylation differences among tissues and the results are summarized in Figure 3. From the 1296 significantly expressed genes, only about one third of them (483 genes) were not shown to have significant changes in methylation between the three tissues. In total, 610 genes were shown to have significant methylation difference between heart and liver, 599 genes were significant between kidney and liver, and 418 genes were significant between heart and kidney. Among them, many genes overlap to be significant in two or three tests. For example, 340 genes showed significant results in comparing both kidney and heart against liver, and 135 genes were significant in all three tests, heart *vs*. kidney, heart *vs*. liver, and kidney *vs*. liver. The list of these 135 genes is shown in additional file 1. The distance of each CpG markers to the transcriptional start site (TSS) was used a covariate to fit into a linear model, but it was not identified as a confounding factor to influence gene expression. In short, the majority of differentially expressed genes showed significant changes in DNA methylation, implying DNA methylation plays an important role in mediating tissue differentiation.

We closely examined the 135 genes that not only have significantly different level of mRNA expression, but also have remarkably different DNA methylation between any pair of the three tissues. We first studied those genes that express very low in kidney, heart and liver (FPKM <1). Their marginal expression may regulate important cellular and biochemical functions, but this requires further elucidation, and and their expression should be more finely tuned. For instance, iodotyrosine deiodinase (*IYD*) was expressed at low levels in all three tissues (0 in heart, 0.02 in kidney, and 0.26 in liver), which was in accordance to its high methylation level (0.81 in heart, 0.61 in kidney and 0.43 in kidney). Its expression in heart and kidney is negligible, but it has low expression in liver. This is consistent with the lower level of DNA methylation in liver. Iodotyrosine deiodinase, also known as iodotyrosine dehalogenase 1 especially expressed in the thyroid, and salvages iodide by catalyzing deiodination of mono- and diiodotyrosine during the biosynthesis of the thyroid hormone thyroxine [24]. The function of IYD in liver remains unknown. Next, we examined other genes that have distinct high expressions in heart, kidney and liver (FPKM>1), which includes 111 genes. This pool of genes may be critical to the development and/or function of kidney, heart and liver. Several genes were selected for further consideration. Transcobalamin I (*TCN1*) is a vitamin B12 binding protein that regulates the absorption, trafficking and secretion. TCN1 abnormality in the liver and kidney has been thought to associate with impairment of these tissues [25]. Its FPKM values were 0, 1.75 and 12.97 in heart, kidney and liver respectively, corresponding to beta values of 0.81, 0.59 and 0.73. Thus, its low expression in heart may be partially regulated by DNA methylation. For another example, serine protease 23 (*PRSS23*), belongs to the peptidase S1 family [26], and is highly expressed in kidney, heart and liver (117.69, 30.86 and 217.32 respectively), and this is consistent with a negative regulatory role for DNA methylation in controlling gene expression as all three tissues show relatively low levels of DNA methylation (0.04 in kidney, 0.07 in heart and 0.06 in liver).

**Figure 4:**
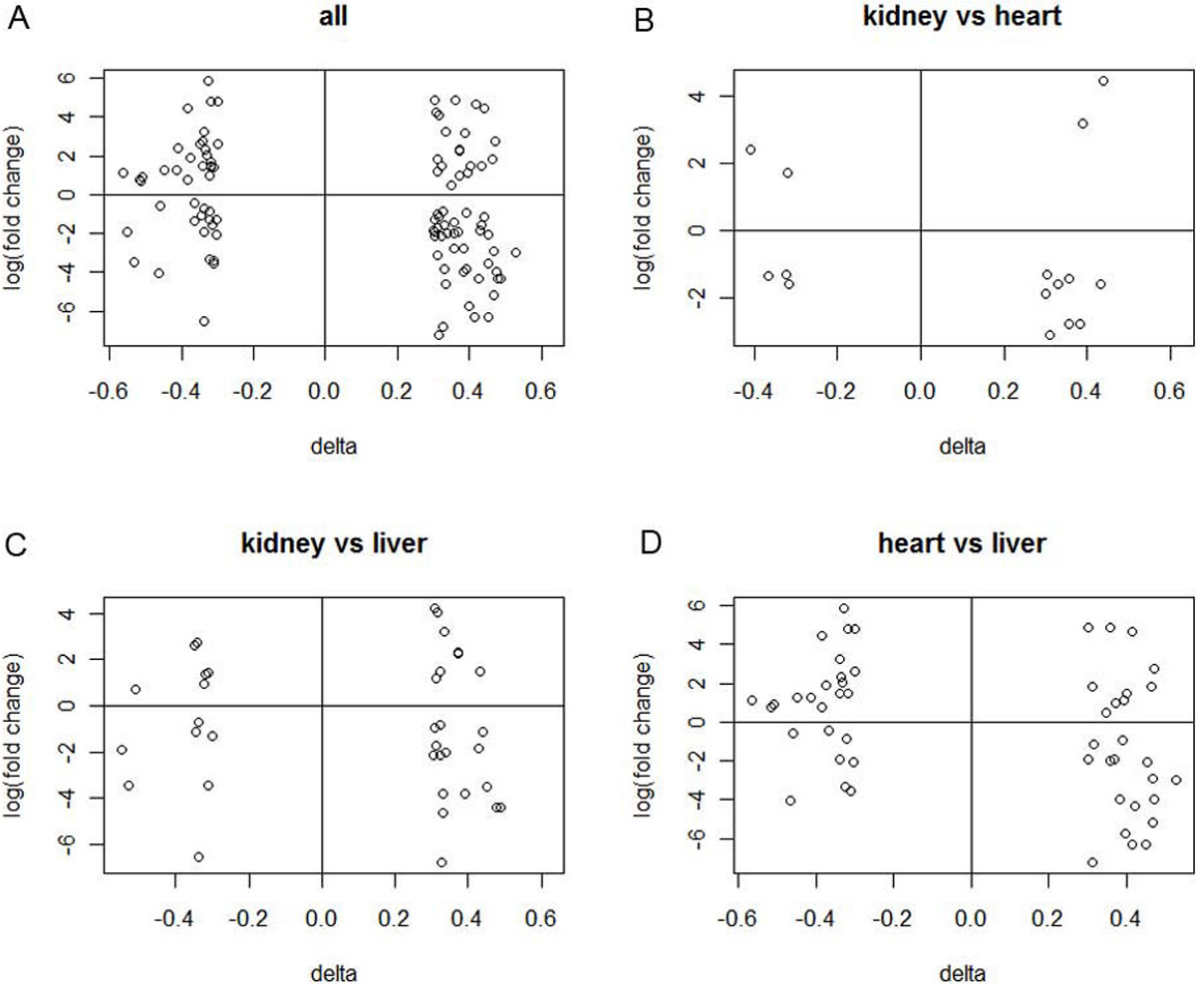
**Expression fold change vs methylation change**. The fold change of expression at natural log scale is used for y axis, and the delta value is the difference in average beta values between two tissues. Only the 1296 differentially expressed genes with delta values greater than 0.3 are plotted. A. The pooled data of all pairs of tissue comparison. BCD. The data for comparison of two tissues.

We next investigated whether DNA methylation is associated with induced or repressed gene expression during tissue differentiation. We selected the genes from the 1296 genes that had sufficient changes in methylation levels between tissues (change of average beta values greater than 0.3) and plotted the changes in methylation and expression levels in Figure 4. The fold change in expression levels was plotted on a logarithmic scale. Although higher methylation levels are associated with both lower and higher expression levels, Figure 4 shows a trend that high methylation is more likely to repress gene expression. For example in Figure 4B of kidney *vs*. heart, 10 genes show higher methylation levels in kidney. Among the 10 genes, 8 show lower expression levels, and only 2 show higher expression levels in kidney than in heart. Therefore, the effect of DNA methylation on gene expression may be bi-directional, nonetheless, higher methylation tends to repress gene expression.

### DNA methylation change correlates with gene expression

**Figure 5:**
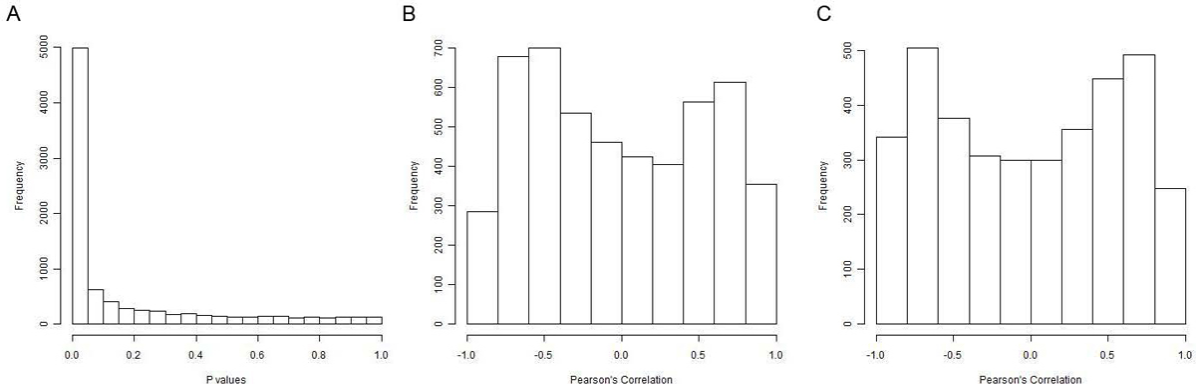
**The correlation of gene expression and DNA methylation**. A. The distribution of p values for correlation of gene methylation and expression. B. Histogram of Person's correlation between gene methylation and expression for 5018 markers within CpG island sites. C. Histogram of Person's correlation between gene methylation and expression for 3669 markers outside CpG island sites.

We further investigated whether the significant changes in methylation will result in a measurable difference in gene expression. To select the genes with significant variation in methylation among the three tissues, we performed analysis of variance (ANOVA) for each gene. To adjust p values for multiple tests, we control false discovery rate (FDR) at 5% and obtained 8687 CpG markers that showed significant variation among the three tissues. We then conducted regression analysis for gene methylation levels and transcriptional levels. Similarly, we adjusted p values by controlling FDR at 5%. Amazingly, most of the 8687 CpG dinucleotides (5735) showed significant correlation between methylation and expression. The p value distribution of regression analysis is shown in Figure 5A. It is clear from this figure that the amount of significant correlations is not generated from random sampling. Thus, we conclude that DNA methylation extensively correlates with gene expression during tissue differentiation.

From the 5735 CpG dinucleotides, 2960 were associated with repressed gene expression, which was only slightly larger than the number of genes (2775) whose methylation levels were positively correlated gene expression. This result further confirms our finding that the effects of methylation on gene expression are bi-directional during tissue differentiation. As HumanMethylation27 markers are located either within or outside CpG island, we are interested to know if the location of markers affects its regulation on expression. Figure 5B-C show the distributions of Pearson's correlations for markers that are located within CpG island (B) or outside of known CpG islands (C). The two distributions are similar with both peaks in positive and negative correlation regions. The peak in negative correlation region is slightly higher than that in positive region, but it is not significant. It indicates a weak trend of increased methylation being associated with decreased gene expression.

We identified several tissue specific genes by sorting these genes whose methylation levels significantly correlates with gene expression. We identified *C4BPA*, expression of 0.03 in kidney, 0.04 in heart and 97.02 in liver, associated with DNA methylation of 0.77, 0.80 and 0.33, respectively. *C4BPA *encodes Complement Component 4 Binding Protein, which is known to be only produced in liver [27]. Other selected genes involving in complement system include *C5AR1, CD55, FGG, and KRT1*. Aquaporin 2 (*AQP2) *was expressed high in the kidney (15.84 in kidney, 0.17 in heart and 0.03 in liver) with corresponding DNA methylation of 0.64 in kidney, 0.77 in heart and 0.87 in liver. We noticed that the values of DNA methylation are relatively high in all three tissues, while the value difference between kidney and heart is 0.14 and that between kidney and liver is as high as 0.24. AQP2, found in the apical cell membrane and intracellular vesicles of the kidney's collecting duct principle cells, is an important vasopressin-regulated water channel, which supports out finding of high correlation between mRNA expression and DNA methylation [28]. These results showed that integrative analysis of genome wide DNA methylation and RNA-Seq transcriptome data can reveal global regulation of gene expression by DNA methylation.

## Discussion

Extensive chromatin remodelling occurs on a global level during development. As embryonic stem (ES) cells differentiate, they lose pluripotency [29,30]. Just as normal stem/progenitor cells are significantly remodelling chromatin during differentiation, it is also important to note that these cells also use DNA methylation, to collaborate with chromatin configuration, to stabilize key gene expression patterns which emerge during normal development and adult tissue cell turnover. For example, localized DNA methylation changes occur at key cytokine target genes during embryonic and adult differentiation and maturation of lymphocytes [31]. We now add data that methylation of CpG dinucleotides also may play a key role in controlling expression of a plethora of genes involved in directing tissue specificity and diverse organ function. It is unclear, however, if this methylation is the cause or simply a marker of gene silencing, and further studies will be required to distinguish between these hypotheses.

The extent that adult tissue-specific stem/progenitor cells retain this global permissive chromatin status is likely tied to the extent with which they retain pluripotency and little is known about DNA methylation differences between cell lineages. This study showed that not only can tissue specific signatures be identified from global DNA methylation patterns, but also the distance at embryonic origin between tissues can be determined. We found that heart and kidney are closer than heart and liver as well as kidney and liver in the distance of global DNA methylation, reflecting heart and kidney has the same embryonic origin, mesoderm, whereas liver develops from endoderm. This finding might be utilized to trace the origin of cell lineage and discover the relationship between cell lineages.

In global genomic packaging, for all types of cells, DNA methylation may be a key component of repressive chromatin which functions to give long-term silencing of transposons, stabilization of silenced genes in the processes of imprinting and x-inactivation [17]. Mammalian females use x-chromosome inactivation to equalize the imbalance of X-chromosome gene expression created by females having two X chromosomes in contrast to the male XY. Dense regions of CpG dinucleotides, termed CpG islands, along the entire X chromosome are also DNA hypermethylated in the embryo [32]. Out of 466 CpG markers that have the highest variability between heart, kidney and liver, 69 markers are located in X chromosome and were highly methylated only in female tissues. All these 69 markers except one are located in CpG islands. The marker that is not in a CpG island is for MSN gene that encodes moesin. Interestingly, even including these X chromosome inactivation genes, the 466 markers still correctly clustered all tissue samples.

Promoter hypermethylation has been mainly associated with gene silencing [33]. However, in this study we found that DNA methylation was either positively or negatively correlated with gene expression. Because the majority of genes that have methylation variation between tissues showed correlation between methylation and transcriptional levels, such number of correlations was so significant that it could not be generated by chance. The number of negative correlations was only slightly larger than that of positive correlations and this difference was not significant. This result suggests two possibilities, either DNA methylation is bi-directional in regulating gene expression or there is a multiple-factor complicated system that monitors gene expression during tissue differentiation. The location of CpG markers has been reported to be a factor to influence the effect of methylation on gene regulation [13]. We have tested locations, such as the distance to transcriptional start site (TSS) or whether inside or outside a CpG island, and failed to find they affect on the correlations of methylation and gene expression. This result need to be further investigated with denser methylation arrays, such as Illumina Infinium 450 K.

Aberrant DNA methylation is also a hallmark of most cancers and the changes begin in the earliest pre-malignant and hyperplastic lesions, and throughout tumor progression, and contributes significantly to the biology of cancer [33-36], but the role of epigenetic/epigenomic changes in other diseases remains an area of active focus of many researchers. In order properly understand the consequence of DNA methylation changes in various human disease states, we need to understand the epigenetic code of normal cells and the role that DNA methylation places in directing tissue specificity. Furthermore, as many disease states such as cancer may present cells with altered differentiation patterns (i.e. evidence for stem cells in cancer) or characteristics of mixed lineages (epithelial to mesenchymal transition) we need to understand the patterns of DNA methylation between different types of normal cells in order to properly gauge the significance of any aberrant findings. We believe that this study will help underscore the importance of methylation changes at CpG dinucleotides in the promoter regions of genes, and serve as a baseline to measure abnormal methylation changes associated with pathological conditions.

## Conclusions

We studied global DNA methylation in three human tissues, heart, kidney and liver, and found that tissues have distinct DNA methylation patterns. It underlines the existence of tissue-specific methylation signatures and implies an important role played by DNA methylation in mediating normal tissue differentiation. By joining with transcriptome data measured by RNA-Seq, we were able to correlate most methylation variation between tissues with gene expression levels. It indicates that DNA methylation influences normal tissue differentiation via regulating gene expression. Further studies need to be carried out to verify our findings in other normal tissues and to investigate the genes and pathways that are involved in this biological process.

## Methods

### DNA methylation quantification

For each sample, DNA methylation level at each CpG site was given in percentage by

$$\beta =\frac{M}{M+U}\cdot 100\%,$$

Where M is the signal strength of methylated CpG given by Illumina HumanMethylation27 array, and U is the signal strength of unmethylated CpG. To ensuring consistent quality between samples, we plotted the beta values between each pair of samples from the same tissues, and the average beta values between each pair of tissues. For each marker, the difference in beta values between tissues was tested with unpaired t test. ANOVA was used to assess the methylation variation among the three tissues.

### RNA-Seq data quantification

The 50 bp paired-end RNA-Seq short reads from Illumina HiSeq 2000 were firstly mapped onto the reference sequences of human genome 19 using Tophat. The reference sequence was downloaded from the genome browser website of the University of California at Santa Cruz on April 13, 2011. The mapped short reads were further processed to assemble transcripts and quantify transcriptional levels using Cufflinks. Transcriptional levels were quantified as Fragments Per Kilobase of exon per Million fragments mapped (FPKM). FPKM values were logarithm transformed for further analysis because the distribution of transcriptional levels skews right. Quantile-quantile plot (QQ plot) between tissues were drawn by R statistical programming language. The significantly differently expressed genes were identified cuffdiff tool within Cufflinks package.

### DNA methylation sample clustering

All 18 samples from 3 human tissues were clustered using hierarchical clustering method. All 27,578 CpG markers were used for clustering. Different dissimilarity and linkage methods were tested and evaluated. The clustering dendrogram was drawn using Pearson's dissimilarity and Ward linkage method.

In order to plot the methylation data in a heatmap, we selected 488 markers whose standard deviation of methylation levels across all18 samples were greater than 0.2. Heatmap of all samples was drawn with samples in rows and markers in column. Hierarchical clustering was used to group samples (rows) using Pearson's dissimilarity and Ward linkage.

Principal components analysis was conducted to investigate the relationship between samples. Singular vector decomposition was used to transform all 27,578 markers into 18 principal components (18 is the number of samples). The samples were plotted on first few principal components for clustering.

### Integrative analysis of DNA methylation and gene expression

Genes that were expressed differentially between any pair of tissues were selected by cuffdiff, a tool package within Cufflinks. In order to find whether these genes had methylation variation as well, we performed t tests for beta values between each pair of tissues, and performed ANOVA to compare beta values among all three tissues. The numbers of genes that show significant differences between tissues both in transcriptional and methylation levels were counted. The distance of CpG markers to TSS was used as a covariate factor for a regression analysis to test if it was a confounding factor in this analysis.

To detect the correlation between DNA methylation and gene expression, we performed ANOVA to select the CpG markers that showed methylation variation among the three tissues. Regression analysis followed to test whether the changes in methylation were correlated with gene expression. All tests were controlled by FDR to adjust for multiple tests. The distribution of p values of regression analysis was drawn to check whether the distribution as uniform. CpG markers were then grouped into their locations, inside or outside CpG islands, and the effects of location on the distribution of Pearson's correlation was tested.

All data processing and analyses were conducted in a Dell PowerEdge R910 worktation.

## Competing interests

The authors declare that they have no competing interests.

## Authors' contributions

LX performed data analysis and generated some of the figures. BW processed the DNA methylation and RNA-Seq data. KZ designed the study and performed statistical analysis. LX, JEO and KZ contributed ideas for data analysis and wrote the manuscript. All authors read and approved the final manuscript.

## Supplementary Material

Additional file 1 Click here for file
