# Supplementary material for: An integrative analysis of DNA methylation and RNA-Seq data for human heart, kidney and liver
Source: BMC Syst Biol. 2011 Dec 23;5(Suppl 3):S4. doi: 10.1186/1752-0509-5-S3-S4 (PMC3287572; doi:10.1186/1752-0509-5-S3-S4)
Supplement: Additional file 1 [file 1752-0509-5-S3-S4-S1.docx]

# Additional file 1

## The list of genes that significant difference between tissues in both expression and methylation

| **Symbol** | **Heart FPKM** | **Kidney FPKM** | **Liver FPKM** | **Heart beta** | **Kidney beta** | **Liver beta** |
| --- | --- | --- | --- | --- | --- | --- |
| CABP1 | 0.776427 | 3.53401 | 0.200824 | 0.131422681 | 0.111556829 | 0.150159225 |
| ABCB11 | 0.539833 | 0.0593874 | 0 | 0.586401035 | 0.708759012 | 0.320970653 |
| HOM-TES-103 | 18.4235 | 10.5026 | 5.40645 | 0.024037458 | 0.316380345 | 0.104360495 |
| FLJ32784 | 0 | 0.221716 | 0.0287253 | 0.659689722 | 0.796715308 | 0.85472229 |
| WFDC10B | 0 | 1.59273 | 0.316076 | 0.656246318 | 0.710715009 | 0.458001808 |
| TMPRSS3 | 0.727359 | 0.187531 | 4.31056 | 0.749060655 | 0.384254177 | 0.667147835 |
| HOM-TES-103 | 18.4235 | 10.5026 | 5.40645 | 0.088376059 | 0.261321823 | 0.150183342 |
| MYO1A | 0.000770987 | 0.304274 | 0 | 0.594455669 | 0.748088106 | 0.849962763 |
| CLEC4M | 38.4774 | 0.165875 | 0.89365 | 0.766815325 | 0.812502224 | 0.629594007 |
| MCEMP1 | 3.93675 | 0.49193 | 19.7599 | 0.175552402 | 0.129706727 | 0.345046159 |
| CD209 | 35.4416 | 9.59118 | 3.52005 | 0.166845869 | 0.376818914 | 0.502513147 |
| IL10RA | 35.2117 | 4.62014 | 12.1258 | 0.67941446 | 0.771486225 | 0.461474628 |
| WNT2 | 1.63302 | 0.378219 | 9.5648 | 0.049559635 | 0.078357373 | 0.209874576 |
| CD8A | 41.5866 | 1.81919 | 10.127 | 0.229908252 | 0.092083241 | 0.158978255 |
| FOXG1B | 1.02493 | 0.0366591 | 0 | 0.082946114 | 0.117733922 | 0.157108009 |
| PSMAL | 0.590757 | 10.807 | 2.26264 | 0.65897962 | 0.556680031 | 0.377692781 |
| CD40LG | 6.7728 | 0.469618 | 1.61745 | 0.743129924 | 0.696412571 | 0.632674833 |
| SPATA18 | 0.583574 | 3.02461 | 14.1292 | 0.752639915 | 0.584737282 | 0.653347192 |
| TSPAN2 | 1.97659 | 28.569 | 5.84117 | 0.05654272 | 0.077093338 | 0.106962129 |
| BMP8A | 0.950287 | 0.178326 | 0.0112026 | 0.097017618 | 0.139819527 | 0.481088615 |
| C19orf21 | 0.0460256 | 0.256137 | 8.05386 | 0.541108568 | 0.222021134 | 0.752726384 |
| TMEM125 | 0.367218 | 2.97042 | 20.1404 | 0.190507603 | 0.106017571 | 0.655960569 |
| EXTL1 | 5.99742 | 0.417873 | 0.0514832 | 0.195631863 | 0.372250249 | 0.512960491 |
| EBF | 15.3158 | 28.1949 | 8.05898 | 0.08007891 | 0.157883031 | 0.261325883 |
| NPY | 17.52 | 98.7172 | 0 | 0.07809286 | 0.334820309 | 0.171302615 |
| PRAME | 0 | 0.198004 | 0.0309721 | 0.379489198 | 0.511472163 | 0.603482424 |
| IRX5 | 0.0604157 | 0.489315 | 1.65405 | 0.076198352 | 0.136168912 | 0.245702375 |
| IRX5 | 0.0604157 | 0.489315 | 1.65405 | 0.047079844 | 0.068769466 | 0.094250535 |
| TH | 2.89682 | 0.742602 | 0.157815 | 0.765584327 | 0.855776747 | 0.688396905 |
| NR1I3 | 0.249654 | 2.71159 | 0.0387293 | 0.736439804 | 0.578053293 | 0.271127467 |
| GATA2 | 3.44922 | 27.233 | 11.0118 | 0.633744126 | 0.549089363 | 0.318166633 |
| S100A3 | 3.08824 | 0.775705 | 11.0607 | 0.558188698 | 0.727400075 | 0.819778076 |
| GPRC5A | 2.22165 | 11.7828 | 128.126 | 0.366865729 | 0.312726132 | 0.246638582 |
| DEF6 | 29.1051 | 3.1146 | 8.21486 | 0.109653798 | 0.352773959 | 0.558191921 |
| FLJ40919 | 1.05426 | 0 | 17.164 | 0.345430455 | 0.737361686 | 0.44659565 |
| LY6D | 0 | 2.6678 | 0.375449 | 0.818447051 | 0.86917505 | 0.927768869 |
| DKK1 | 0.217664 | 7.3454 | 1.20314 | 0.067302156 | 0.100995283 | 0.143619217 |
| PCBP3 | 9.79136 | 2.14043 | 0.934496 | 0.608667435 | 0.726080078 | 0.476335565 |
| SGCA | 4.9934 | 44.5602 | 15.9989 | 0.231457881 | 0.388948785 | 0.326295624 |
| CCL14 | 277.401 | 118.705 | 42.1097 | 0.429218814 | 0.605779535 | 0.677731074 |
| SH3BP2 | 33.7843 | 3.63078 | 15.6999 | 0.279749064 | 0.450493682 | 0.66500636 |
| TH | 2.89682 | 0.742602 | 0.157815 | 0.816741823 | 0.929908556 | 0.64804297 |
| FCRL3 | 8.64818 | 0.163318 | 0.907831 | 0.638341324 | 0.698953383 | 0.538920025 |
| SH3BP2 | 33.7843 | 3.63078 | 15.6999 | 0.139736736 | 0.358904649 | 0.656153365 |
| CD3E | 127.898 | 4.4681 | 24.8457 | 0.193486412 | 0.269560852 | 0.366837006 |
| ANGPT1 | 4.63151 | 13.8765 | 36.0387 | 0.388358905 | 0.25477136 | 0.303940154 |
| PI3 | 0.273057 | 2.06148 | 68.7415 | 0.445298009 | 0.582509683 | 0.314182447 |
| IL2 | 1.02079 | 0 | 0.0966061 | 0.807396493 | 0.769946464 | 0.693847162 |
| MYO1A | 0.000770987 | 0.304274 | 0 | 0.561271778 | 0.653957426 | 0.778844077 |
| KLK11 | 1.10164 | 6.08573 | 19.9972 | 0.550931806 | 0.481706615 | 0.348064821 |
| FAT2 | 0.0171411 | 1.48041 | 0.125462 | 0.309022228 | 0.74858121 | 0.482768726 |
| MATN2 | 45.0541 | 115.418 | 28.2721 | 0.69002169 | 0.548631667 | 0.341692148 |
| IL5RA | 9.1428 | 0.117313 | 1.32646 | 0.553498065 | 0.824025122 | 0.717194852 |
| FOXG1B | 1.02493 | 0.0366591 | 0 | 0.070490422 | 0.093675804 | 0.115629677 |
| TNR | 0.097692 | 0 | 0.41707 | 0.673757525 | 0.75669325 | 0.544210095 |
| NKX6-1 | 0.619901 | 0 | 0.0996529 | 0.080545595 | 0.097875805 | 0.126138112 |
| UPB1 | 0 | 0.105868 | 0.344579 | 0.689798836 | 0.568008162 | 0.409713603 |
| BPIL1 | 0.0459702 | 0.312274 | 24.4867 | 0.765316589 | 0.83887428 | 0.351214814 |
| CAV2 | 28.5408 | 65.8187 | 158.794 | 0.327903152 | 0.443144047 | 0.274568092 |
| COL7A1 | 0.662228 | 4.12416 | 1.80275 | 0.124604366 | 0.07116615 | 0.21922057 |
| ITGAX | 4.08991 | 1.06235 | 11.8107 | 0.082088875 | 0.054129057 | 0.16118401 |
| CLDN16 | 0.0449289 | 0.573888 | 1.91172 | 0.835055025 | 0.709041342 | 0.862564261 |
| UNQ3033 | 0.123303 | 0 | 0.547423 | 0.369367661 | 0.474714127 | 0.312665788 |
| NPY | 17.52 | 98.7172 | 0 | 0.079566294 | 0.326223255 | 0.136779255 |
| C21orf13 | 0 | 0.452506 | 1.98825 | 0.363004621 | 0.430107106 | 0.566174856 |
| AQP2 | 0.166219 | 15.8803 | 0.0268801 | 0.773650494 | 0.658791945 | 0.87056063 |
| SULT1A2 | 0.29651 | 0.842693 | 2.2952 | 0.743920436 | 0.560254789 | 0.385951392 |
| FAM78A | 12.1577 | 0.75791 | 2.74308 | 0.01739724 | 0.033680741 | 0.335747677 |
| EHF | 0.314145 | 23.9703 | 10.715 | 0.508810383 | 0.388059383 | 0.223055621 |
| CD48 | 174.275 | 5.49781 | 57.8282 | 0.749196314 | 0.695742647 | 0.607273696 |
| ADCY5 | 5.20614 | 16.1559 | 2.04044 | 0.116043799 | 0.165356294 | 0.306012824 |
| IL8RA | 4.90771 | 0.608467 | 16.1641 | 0.512469961 | 0.661460847 | 0.439243202 |
| UBASH3A | 3.18397 | 0.0940626 | 0.811096 | 0.537155258 | 0.614066002 | 0.407681432 |
| CYP4F2 | 0 | 0.651785 | 0.199748 | 0.852266419 | 0.750842009 | 0.439890731 |
| S100A2 | 1.25386 | 29.2291 | 87.3905 | 0.498557653 | 0.275293546 | 0.621691214 |
| TRIM63 | 0.660622 | 7.59578 | 0.0480645 | 0.417158446 | 0.650743651 | 0.516038667 |
| MYH6 | 0.120574 | 0.0251106 | 0 | 0.479894446 | 0.91282233 | 0.794481036 |
| DEF6 | 29.1051 | 3.1146 | 8.21486 | 0.192514358 | 0.401839933 | 0.605032341 |
| LTF | 8.19168 | 31.4082 | 152.819 | 0.426042297 | 0.263928076 | 0.189704374 |
| GGTLA1 | 34.444 | 17.0091 | 5.18406 | 0.324992166 | 0.557934181 | 0.700463062 |
| CDX1 | 0.33132 | 0.0657537 | 0 | 0.804161685 | 0.740751104 | 0.683949263 |
| SAA1 | 159.335 | 19.5224 | 61.4834 | 0.686221789 | 0.752750688 | 0.313275646 |
| PRG2 | 0 | 0.0145761 | 2.97742 | 0.086538639 | 0.116944581 | 0.194875769 |
| WNT2 | 1.63302 | 0.378219 | 9.5648 | 0.040618778 | 0.050562484 | 0.08316777 |
| ST6GAL1 | 118.046 | 22.8379 | 46.471 | 0.095863393 | 0.267294686 | 0.604209941 |
| TFPI | 135.462 | 48.689 | 357.74 | 0.637858607 | 0.732113881 | 0.394169528 |
| CLEC4G | 51.5441 | 3.24347 | 0.586736 | 0.497611333 | 0.623454162 | 0.659234317 |
| DLX1 | 0.165225 | 1.09592 | 0 | 0.054058076 | 0.165175124 | 0.27379564 |
| CD8A | 41.5866 | 1.81919 | 10.127 | 0.053057504 | 0.038945533 | 0.070159965 |
| LTF | 8.19168 | 31.4082 | 152.819 | 0.495981325 | 0.353493978 | 0.24728791 |
| UPB1 | 0 | 0.105868 | 0.344579 | 0.881623772 | 0.664141201 | 0.483086104 |
| TNNT2 | 2.70375 | 29.8615 | 8.79026 | 0.556326543 | 0.809819745 | 0.691034093 |
| GGTLA1 | 34.444 | 17.0091 | 5.18406 | 0.413095062 | 0.46958112 | 0.595427856 |
| FOXM1 | 3.81124 | 0.937862 | 0.35407 | 0.167312733 | 0.062607098 | 0.046039325 |
| GPC2 | 1.35371 | 0.638972 | 0.135171 | 0.060483891 | 0.120302052 | 0.218835257 |
| HABP2 | 0.00355502 | 0 | 0.33581 | 0.669191566 | 0.456298075 | 0.285827127 |
| LBP | 3.44293 | 0.271003 | 0.0257617 | 0.511021229 | 0.343561607 | 0.208282403 |
| TIMD4 | 13.8327 | 2.49468 | 0.0424611 | 0.471923145 | 0.705006872 | 0.390295614 |
| CKM | 4.1896 | 1.13873 | 0.0115565 | 0.539594146 | 0.753738135 | 0.86643354 |
| PACAP | 174.824 | 1.83718 | 17.0688 | 0.633418651 | 0.800956274 | 0.898993407 |
| DSCAML1 | 0.0985875 | 0.412637 | 1.58904 | 0.062880753 | 0.053882379 | 0.13801008 |
| TCN1 | 0 | 1.79284 | 12.9745 | 0.736166913 | 0.812998276 | 0.59495535 |
| MLF1 | 4.31433 | 10.0153 | 22.9905 | 0.032717756 | 0.046727769 | 0.174939327 |
| GLP2R | 2.76287 | 14.6647 | 0.546859 | 0.459051453 | 0.63765006 | 0.513564325 |
| C6orf71 | 0 | 0.0201024 | 0.262979 | 0.80771716 | 0.608601885 | 0.427543321 |
| ZNF323 | 1.04018 | 6.9286 | 3.38477 | 0.656676683 | 0.74738255 | 0.855258976 |
| TIAM2 | 1.65677 | 4.32952 | 0.521315 | 0.918723758 | 0.858018529 | 0.700015116 |
| RAB34 | 30.282 | 92.2446 | 45.4746 | 0.158632881 | 0.104848456 | 0.61668288 |
| SLC22A16 | 0.0985961 | 0 | 0.961385 | 0.040878743 | 0.07646222 | 0.290855062 |
| TCP11 | 0 | 0.0829339 | 0.370637 | 0.536713251 | 0.459199473 | 0.698285001 |
| SLC5A7 | 0.0248399 | 1.26804 | 0.160475 | 0.781493296 | 0.869929076 | 0.638451884 |
| LCN2 | 0.424275 | 16.8216 | 144.26 | 0.685196465 | 0.596776731 | 0.442086453 |
| GGT6 | 0 | 6.34645 | 2.40714 | 0.794130627 | 0.501535043 | 0.711263924 |
| GDF3 | 6.40039 | 0.286473 | 0 | 0.283411216 | 0.218517031 | 0.41742118 |
| WFDC10B | 0 | 1.59273 | 0.316076 | 0.816949846 | 0.895564208 | 0.789934174 |
| P2RY10 | 40.314 | 0.210007 | 4.56291 | 0.465010689 | 0.567054487 | 0.306956116 |
| PRSS23 | 30.8631 | 116.394 | 217.321 | 0.062669759 | 0.04745825 | 0.073881185 |
| CDX1 | 0.33132 | 0.0657537 | 0 | 0.955033097 | 0.968285078 | 0.921631368 |
| SYT8 | 0.0121146 | 7.75575 | 1.84754 | 0.800532658 | 0.562352038 | 0.775145561 |
| ARHGEF4 | 0.365578 | 8.76085 | 1.98999 | 0.769722246 | 0.886372801 | 0.564156746 |
| FAM83A | 0 | 2.49237 | 0.584149 | 0.758378725 | 0.849105968 | 0.916364752 |
| CD3D | 79.6811 | 2.06014 | 20.5777 | 0.69271736 | 0.838951795 | 0.772626153 |
| UNC45B | 0.265992 | 0.047859 | 0.754256 | 0.548896957 | 0.815393507 | 0.786201681 |
| MGC35295 | 0.0831521 | 0 | 17.1685 | 0.624288319 | 0.721476252 | 0.517780481 |
| FLJ46481 | 0.00985693 | 0 | 0.0437517 | 0.372617374 | 0.652461328 | 0.511216412 |
| FCRL3 | 8.64818 | 0.163318 | 0.907831 | 0.46539657 | 0.635728835 | 0.323877878 |
| S100A14 | 0.249964 | 16.0625 | 43.745 | 0.928377244 | 0.681218334 | 0.460441631 |
| NME5 | 0.27704 | 2.31577 | 9.22183 | 0.210851564 | 0.124235446 | 0.360271749 |
| MMP7 | 0.132675 | 19.128 | 4.70458 | 0.550363138 | 0.348942142 | 0.667110677 |
| ADORA3 | 22.6921 | 5.22384 | 0.0767424 | 0.391925398 | 0.445252891 | 0.50684686 |
| INA | 0.445355 | 1.79785 | 0.0301401 | 0.050517785 | 0.07057694 | 0.224547605 |
| ITGAX | 4.08991 | 1.06235 | 11.8107 | 0.168314942 | 0.097373315 | 0.210528808 |
| FLJ25422 | 3.77356 | 12.0984 | 0.880501 | 0.861241179 | 0.691450823 | 0.458354767 |
| PYGM | 4.65159 | 21.4261 | 1.54091 | 0.078081641 | 0.294271104 | 0.64263458 |
| SHRM | 0.34379 | 4.43182 | 10.0118 | 0.2139669 | 0.256210205 | 0.49450965 |
